# Factors influencing person-centred care: Speech-language pathologists and audiologists perspective

**DOI:** 10.4102/ajod.v14i0.1589

**Published:** 2025-03-25

**Authors:** Faheema Mahomed-Asmail, Louise Metcalfe, Marien A. Graham, Renata Eccles

**Affiliations:** 1 Department of Speech Language Pathology and Audiology, Faculty of Humanities, University of Pretoria, Pretoria, South Africa; 2 Department of Early Childhood Education, Faculty of Education, University of Pretoria, Pretoria, South Africa

**Keywords:** person-centred care, socioeconomically diverse, facilitators, barriers, speech-language pathology, audiology

## Abstract

**Background:**

Person-centred care (PCC) is a fundamental aspect of healthcare, and its implementation is primarily based on clinicians’ initiation and sustained efforts and the availability of resources. Recent PCC literature has primarily focused on high-income settings, raising concerns about the feasibility of PCC implementation in low- and middle-income countries.

**Objectives:**

This study examined speech-language pathologists’ (SLPs) and audiologists’ (AUDs) perceptions of barriers and facilitators towards implementing PCC in the diverse South African context, particularly how their demographic factors influence these perceptions.

**Method:**

A national cross-sectional e-survey pooled 103 clinicians who were providing speech-language pathology and audiological services in South Africa. The e-survey included questions on participants’ demographics, working environment and a seven-point Likert scale rating 10 components that influence PCC.

**Results:**

Clinicians scored personal factors (64.7%), followed by their relationships with different professionals (54.9%) as the most facilitating factors for achieving PCC. The most significant perceived barrier was resources, including time and finances (59.8%), followed by the client perspectives (53.9%). Significant associations were found between the components influencing PCC and clinicians’ qualifications, work sectors and populations served.

**Conclusion:**

The collective findings of this study highlighted the multifaceted nature of PCC implementation within a diverse healthcare context. Client perspectives need to be considered while leveraging clinician attributes and fostering supportive workplace environments for the successful adoption of PCC.

**Contribution:**

This study contributes to literature of PCC implementation and has captured how the perceptions of speech-language therapists (SLPs) and AUDs call for tailored approaches in diverse healthcare contexts.

## Introduction

Person-centred care (PCC) is a concept that describes a model of care that changes the role of the client within the healthcare system. Person-centred care is the shift from a traditional biomedical model to a biopsychosocial model where more equitable power roles between clients and clinicians exist (American Geriatrics Society Expert Panel on Person-Centred Care 2016). This results in management that is tailored to clients and, as such, has functional benefits to their lives (Byrne, Baldwin & Harvey 2020). This shift is a global movement in response to the acknowledged improvement in safety and quality of service delivery for clients, clinicians and larger communities when PCC is implemented (Engle et al. 2021). Specific benefits of adopting a PCC approach include improved access to care, health literacy and higher client and staff satisfaction (World Health Organization [WHO] 2016). However, the implementation of PCC is dependent on various factors, including clinician, client and environmental factors (Danermark 2014).

Among the key considerations in PCC is its role in addressing the needs of individuals with disabilities, who often face barriers to equitable healthcare access (Wakeham et al. 2017). Person-centred care emphasises tailoring services to individual needs, which is particularly relevant for people with disabilities, as it promotes inclusivity, shared decision-making and the provision of care that considers their functional abilities and social contexts (WHO 2016). Although a collaborative model with clients, PCC initiation, implementation and adherence are dependent on the efforts of clinicians providing healthcare services. Identified clinician-specific factors that facilitate the delivery of PCC-related services include training and education, access to measurement and evaluation tools and supportive work environments for health professionals (Levey et al. 2019; Mahomed-Asmail et al. 2024; Moore et al. 2016). Clinicians providing healthcare services have mentioned that variations in what is considered PCC are a perceived barrier to its implementation (Forsgren, Åke & Saldert 2022; Grenness et al. 2014; Moore et al. 2016). Person-centred care is regarded by clinicians as challenging to define and implement, especially in diverse and demanding contexts (Cooper, Smith & Hancock 2008; Grenness et al. 2014; Stewart et al. 2013). Definitions exist, including an approach that respects clients’ preferences and values, involves family and friends, reinforces shared decision-making and goal setting and prioritises information exchanges (Person-Centred Hearing Network n.d.). There is, however, currently no universally accepted definition that leaves the concept open to interpretation by those tasked with implementing it, namely clinicians, management structures and policymakers (Byrne et al. 2020).

Additional reported barriers that impede implementation are clinicians’ and professional team members’ adherence to the traditional biomedical model of care as well as clinicians’ own personal beliefs, values and culture (Bolster & Manias 2010; Choy-Brown 2021; Manchaiah et al. 2014; Moore et al. 2016; Sladdin et al. 2017). Time has also been noted to limit clinicians’ capacity to implement PCC. Following a PCC approach is typically time intensive to allow clinicians to get to know their clients and determine specific needs and requirements (Gluyas 2015; Singh et al. 2017). Current billing systems do not always cover the extended time spent with clients (Choy-Brown 2021). This situation gives rise to conflicts between financial interests and clients’ optimal well-being, as healthcare services, especially in the private sector, operate more as business entities (Choy-Brown 2021). Limited time and capacity further impact the development of the therapeutic alliance between clinician and client. This component is essential for breaking down traditional and preconceived power roles within the dyadic relationship (Beck & Kulzer 2018).

Across current PCC research, high-income settings have been the primary focus, raising questions about the feasibility of its implementation in low- and middle-income countries (LMICs). In LMICs like South Africa, clinicians have to overcome additional challenges that are rare in high-income settings when delivering services. An example of the complex challenges in South Africa is the Quadruple Burden of Disease (QBD) – a multidimensional challenge arising from biological, environmental and economic factors (Black et al. 2017). The QBD includes a range of health issues prevalent in South Africa, such as human immunodeficiency virus/acquired immunodeficiency syndrome (HIV/AIDS), tuberculosis, violence, injury, maternal and child health and the surge of non-communicable diseases (Basu 2018). This adds additional pressure to an already complex socioeconomic and culturally diverse landscape. As a result, healthcare clinicians’ focus is not on implementing a PCC approach but managing clients’ health, access and safety.

A recent investigation by Mahomed-Asmail et al. (2023) found that South African clinicians have a high preference towards person centredness. As part of a broader project investigating PCC implementation in South Africa, the qualitative component of this research (Mahomed-Asmail et al. 2024) revealed that clinicians perceive sociodemographic factors – particularly language and cultural diversity, as well as resource constraints – as significant barriers to PCC. These findings highlight the need to further explore how such factors influence clinicians’ ability to adopt PCC in practice. Building on this qualitative work, this article aimed to answer the following questions: *How do speech-language pathologists and audiologists perceive the barriers and facilitators to implementing person-centred care (PCC) in the diverse South African context, and how do their demographic factors influence these perceptions?*

## Method

### Study design and participants

The study employed a cross-sectional survey design (Wisdom & Creswell 2013) and followed the Checklist for Reporting of Survey Studies (CROSS) (Online Appendix 1) (Sharma et al. 2021). A part of the larger project, an e-survey (Online Appendix 2) was distributed to registered communication-related healthcare practitioners providing speech-language pathology and/or audiology services in South Africa, including audiologists (AUDs), speech-language pathologists (SLPs), dually qualified SLPs and AUDs and acousticians. A convenience sampling method was used, whereby the survey was distributed through online social media platforms (Facebook™, LinkedIn™, WhatsApp™), professional associations (South African Speech Language and Hearing Association, South African Association of Audiologists) and by forwarding to the researchers’ networks of colleagues and collaborators practising in South Africa (Mahomed-Asmail et al. 2024).

### Instrument and procedures

The e-survey was made available to participants using Qualtrics™ (Provo, UT) for 3 weeks between October and November 2022. The e-survey was set up to allow only one attempt. It consisted of (1) biographic information, (2) a 7-point Likert scale to rate 10 components relating to possible barriers and facilitators involved in providing PCC (adapted from Danermark 2014) and (3) four open-ended questions further probing their perspectives. Results from the third section are not included in this article because of the quantitative nature and depth of analysis; the findings have been published in a parallel publication (Mahomed-Asmail et al. 2024).

The demographic section of the e-survey collected data on age, sex, current profession, number of years working in the field, employee position, work sector (public, private or academic) and the linguistic and culture background of both clinician and their clients served. Section two followed with 10 components related to barriers and facilitators clinicians face towards PCC. The 10 components were developed by Danermark based on literature and surveyed clinicians’ experiences (Danermark 2014). The barrier and facilitator factors detailed as part of the 10 components include personal (clinician-related), client perspectives, staff knowledge, workplace culture, resources, tools, relationships between different professions, regulations/rules, management and sales focus (Online Appendix 2).

In order to ensure validity and reliability, pilot testing of the e-survey was conducted with five clinicians (one academic, one AUD practising in private, one in the public sector, one speech-language therapist (SLT) in private and one in the public sector). The survey was electronically shared with the five clinicians who completed the survey and provided feedback on aspects that need to be adjusted to improve the clarity and understanding of the survey. Based on the feedback, definitions for each of the 10 components were adjusted to provide participants with better insight into each component. The Likert scale was also expanded from a 5-point Likert scale (a hinder and help scale ‘help++’, ‘hinder--’) (Danermark 2014) to a modified 7-point Likert scale, ranging from 1 (extreme barrier) to 7 (extreme facilitator). The same five clinicians were then asked to review the amended survey and provide any additional feedback, of which there was none.

### Data analysis

The data were analysed with the Statistical Package for Social Sciences (SPSS v.27.0) using descriptive and inferential statistics. Descriptive statistics, including frequency distributions and percentages, were used to summarise the data. After investigating the frequency distributions of data collected from Section 2 of the survey, it was decided to collapse the categories of the 7-point Likert scale back to a 5-point Likert scale because of sparse data in many of the categories. For cross-tabulations of nominal variables (with three or more categories), the independent columns proportions *z*-test was applied to detect significant differences between the categories of a variable (columns) in terms of participants’ perspectives of barriers and facilitators (rows). For example, when exploring the differences in the perspectives between (1) AUDs, (2) SLPs and (3) dually qualified clinicians, for a specific perspective (e.g. item is viewed to be an extreme barrier), the proportions z-test compared the responses between these categories, thus producing three *p*-values for these three pairwise comparisons. If the *p*-value was less than 0.05, the responses differed significantly between the pair being compared.

Correlations were also run, with Spearman correlation (*r*_*s*_), when two variables were ordinal and the point-biserial correlation (*r*_*pb*_) when one variable was binary and the other ordinal. As an example of r_*s*_, clinicians’ age (ordinal) and whether tools were seen as a barrier or facilitator. If the correlation was negative, then the older clinicians (higher age) tended to view tools as a barrier (lower end of Likert scale) and, if positive, the older the clinician (higher age) tended to view tools as a facilitator (upper end of Likert scale). As an example of r_*pb*_, clinicians’ home language (binary, 0 = English, 1 = Other) and tools. If the correlation was negative, then English-speaking clinicians (coded lowest) viewed tools more as a facilitator (higher end of Likert scale) and, if positive, English-speaking clinicians (coded lowest) viewed tools more as a barrier (lower end of Likert scale).

### Ethical considerations

The study received ethical approval from the Institutional Research Board, Research Ethics Committee (ResEthics), Faculty of Humanities, University of Pretoria (No. HUM024/0422). Participants provided written informed consent prior to completing the e-survey, and no identifying information was collected in order to ensure anonymity.

## Results

### Participant demographics

A total of 127 surveys were initially collected. After excluding responses lacking consent or those incomplete despite consent, 103 responses were retained. The removal of surveys with incomplete data was necessary as those respondents exited the survey prematurely, leaving substantial sections unanswered. Of the 103 responses, 91.3% were females and 42.7% were AUDs (Table 1). Only one acoustician participated, and the data collected from this submission were included under the AUD category because of sparsity. A few participants (14.6%) were based in academia and were involved in research, clinical training/supervision or teaching, with some participants completing their postgraduate studies full time.

**TABLE 1:** Participants’ demographics and client population served (*N* = 103).

Demographics	*n*	%
**Gender**		
Female	94	91.3
Male	8	7.7
Other	1	1.0
**Age (years)**		
< 25	33	32.0
26–35	36	35.0
36–45	18	17.5
46–55	10	9.7
> 56	6	5.8
**Current profession**		
Audiologist	44	42.7
Speech-language therapist	34	33.0
Dual†	25	24.3
**Client age profile (years)** ‡		
0–5	76	73.8
6–18	79	76.7
19–65	70	68.0
> 65	61	59.2
**Clinicians’ home language**		
English	41	39.8
Other South African languages (excluding English)	62	60.2
**Clients’ home language**		
English	40	38.8
Other South African languages (excluding English)	63	61.2
**Number of clients seen daily**		
1–5	40	38.8
6–10	50	48.6
11–15	11	10.7
> 15	2	1.9
**Employment and healthcare sector** ‡		
Private practice	52	50.5
Academia	15	14.6
Public sector	41	39.8
Community service‡	21	20.4
Independent practitioner‡	20	19.4
**Caseload distribution‡**		
**Private practice**		
In-patient	-	28.0
Out-patient	-	72.0
School-based	-	38.0
Clinic-based	-	32.0
Other	-	6.0
**Public practice**		
In-patient	-	31.7
Out-patient	-	39.0
School-based	-	17.1
Clinic-based	-	12.2
Other	-	9.8

† , Participants were qualified and practising as both speech-language therapists and audiologists;

‡ , Multiple response options were allowed; therefore, the total number of responses is larger than the sample size (103).

Most participants were between the ages of 26 and 35 years (35.0%), with just over half practising in the private sector (50.5%). The majority of clinicians’ daily caseload ranged between 6 and 10 clients (48.6%), seen predominantly as outpatients. The majority of the clinicians (60.2%) and their clients (61.2%) indicated English was not their home language, but rather one of the other 11 South African official languages, with Afrikaans being the most common home language of clinicians (48.5%) and clients (27.2%) followed by isiZulu for clients (12.6%). Participants also indicated that their language (49.5%) and culture (32.0%) typically sometimes matched those of their clients. The number of clients seen daily, the home language and culture of the clinicians and the culture and language of clients showed no significant associations across the various factors. More than half (59.2%) of the participants indicated that they followed a PCC approach to service delivery.

### Facilitators of person-centred care

**FIGURE 1:**
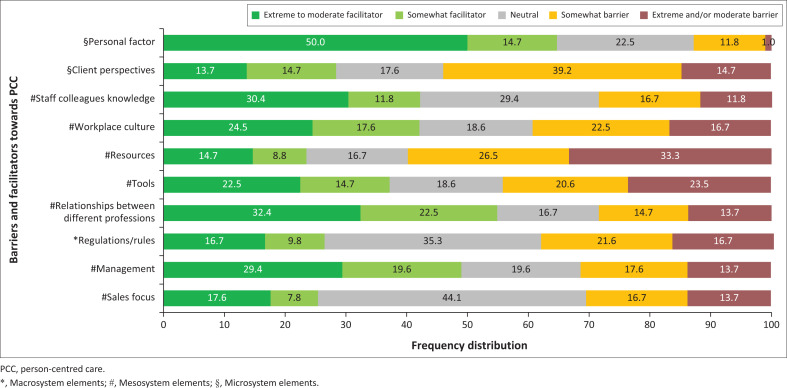
Perceived facilitators and barriers towards person-centred care.

Participating clinicians identified their personal factors (64.7%) as the most facilitating component to implementing PCC, followed by their relationships with different professionals (54.9%) (Figure 1).

Point-biserial correlations showed that participants working in academia tended to view client perspectives (*r_pb_* = 0.20, *p* = 0.039) and workplace culture (*r*_*pb*_ = 0.28, *p* = 0.004) significantly more as facilitators than barriers (Table 2). Clinicians who predominantly worked with adult populations significantly identified sales as a facilitator rather than a barrier (19–65 years, *r*_*pb*_ = 0.25, *p* = 0.011). On the other hand, clinicians serving young clients (0–5 years) only had one significant correlation when considering all 10 factors; they perceived tools significantly more as a facilitator than a barrier (*r*_*pb*_ = 0.21, *p* = 0.030).

**TABLE 2:** Seven of the factors with correlations to demographic variables.

Demographic variable	Client perspective	Workplace culture	Resources	Tools	Regulation and rules	Management	Sales focus
**Clinician demographics**							
Clinicians age	−0.02	0.07	0.16	−0.08	−0.22*	−0.00	0.10
Years working in the field	0.07	0.05	0.13	−0.18	−0.25*	−0.01	0.10
No. of clients seen daily	−0.15	0.06	0.04	0.09	−0.03	−0.03	0.01
**Healthcare sector**							
Private	0.13	−0.04	0.17	0.15	−0.06	0.02	−0.12
Public	−0.20*	−0.16	−0.31*	−0.12	0.02	−0.02	0.15
Academia	0.20*	0.28*	0.19	−0.01	0.02	0.09	−0.08
**Client age (years)**							
0–5	0.05	0.05	−0.06	0.21*	0.10	0.09	−0.14
6–18	−0.07	0.01	−0.14	−0.03	0.10	0.04	−0.05
19–65	−0.21*	−0.27*	−0.10	−0.02	0.03	−0.22*	0.25*
> 65	−0.26*	−0.30*	−0.27*	0.00	0.12	−0.07	0.10

* , Statistically significant at *p* < 0.05.

### Barriers towards person-centred care

The most significant perceived barrier was resources, which included time and finances (59.8%), followed by client perspectives (53.9%). The proportions z-test revealed that AUDs perceived client perspectives as ‘somewhat a barrier’ (62.8%) significantly more than SLPs (26.5%, *z* = 2.807, *p* = 0.005) and dually qualified participants (16.0%, *z* = 3.291, *p* = 0.001). Dually qualified practitioners indicated regulations and rules as ‘somewhat a barrier’ (40.0%) significantly more than SLPs (29.4%, *z* = 2.005, *p* = 0.045) but not significantly more than AUDs (37.2%, *z* = 0.000, *p* = 1.000).

Point-biserial correlations showed that participants from the public sector tended to perceive *client perspectives* (*r*_*pb*_ = −0.20, *p* = 0.047) and *resources* (*r*_*pb*_ = −0.31, *p* = 0.002) significantly more as barriers rather than facilitators (Table 2). Older participants (*r*_*pb*_ = −0.22, *p* = 0.025) and participants with less experience (*r*_*pb*_ = −0.25, *p* = 0.011) identified *rules and regulations* significantly more as barriers rather than facilitators. When considering clients served, clinicians who indicated they served adults perceived *client perspectives* (19–65 years, *r*_*pb*_ = −0.21, *p* = 0.036; > 65 years, *r*_*pb*_ = −0.26, *p* = 0.007), *workplace culture* (19–65 years, *r*_*pb*_ = −0.27, *p* = 0.005; > 65 years, *r*_*pb*_ = −0.30, *p* = 0.002), *resources* (> 65 years, *r*_*pb*_ = −0.27, *p* = 0.007) and *management* (19–65 years, *r*_*pb*_ = −0.22, *p* = 0.026) significantly more as barriers than facilitators.

## Discussion

The present study provides an exploration of the perceptions of SLPs and AUDs in South Africa regarding the facilitators and barriers associated with implementing PCC and the influence demographic factors had on these perceptions. The diverse sample of 103 clinicians, predominantly females (94.0%), shed light on factors influencing the delivery of PCC in a socioeconomically and linguistically diverse setting faced with the QBD.

More than half of the respondents (59.2%) indicated their adherence to a PCC approach in service delivery, despite possible challenges to implementation, including that their home language and cultural background did not consistently align with those of their clients. The mismatch between clinician and client language and culture was a reported concern by clinicians (Mahomed-Asmail et al. 2024); however, contrary to expectations, there were no statistically significant associations found between home language, culture and the 10 components examined in this study. An association was expected, given the acknowledged and reported influence that cultural, linguistic and socioeconomic disparities can have on PCC implementation (Anderson et al. 2003; Mahomed-Asmail et al. 2023, 2024).

Clinicians’ motivation to adopt a PCC approach in their service delivery has been substantiated by prior research (Bellon-Harn et al. 2017; Laplante-Lévesque et al. 2014; Mahomed-Asmail et al. 2023, 2024). Consistent with this premise, clinicians’ personal factors emerged as the most influential facilitator of PCC implementation. These personal factors encompassed the clinicians’ passion, commitment, vision, courage and perseverance (Danermark 2014), all of which are integral clinical attributes facilitating an understanding of clients’ emotional states, individual needs and readiness for change. These attributes foster a supportive environment that ensures a collaborative decision-making process (Ekberg, Grenness & Hickson 2014; English 2022 Grenness et al. 2014; Moore et al. 2016). Surprisingly, the reported adherence to providing PCC was not significantly associated with personal factors. Clinician-related variables such as age and years of experience were, however, a significant influence on clinicians’ perceptions of rules and regulations, encompassing protocols, legislation, regulations, practice guidelines, position statements and standards. Older participants (> 46 years) and those with limited experience (< 25 years) identified rules and regulations as a substantial barrier to PCC, which are in line with their qualitative responses (Mahomed-Asmail et al. 2024). This phenomenon may arise from younger and older clinicians, those with less and more experience, respectively, grappling with the tension between what they ‘should’ do and what they ‘must’ do, underscoring their constrained autonomy to practise PCC within the confines of the established system standards (Byrne et al. 2020). Notably, a significant association was also observed between dually qualified clinicians and their views of rules and regulations. This association can likely be attributed to the requirement for dually qualified clinicians to navigate and provide services within the frameworks of two distinct scopes of practice (Health Professional Council of South Africa [HPCSA] 2011, 2017).

In a recent review by Byrne et al. (2020), the theme of ‘the power to practise PCC’ emerged as a significant element within the field of nursing. This theme encompassed various factors, including workplace culture, leadership, policy and practice, organisational systems, environmental workload and ward culture, which either facilitated or hindered the implementation of PCC (Byrne et al. 2020). When assessing workplace culture and management in this study, clinicians’ perceptions were evenly divided, with both aspects receiving slightly more positive evaluations than negative ones, particularly among academics, who regarded workplace culture as a significant facilitator for PCC implementation. Academics also contributed to a heightened awareness of the evolving healthcare landscape that is increasingly oriented towards the principles of PCC (Fernandes et al. 2022; WHO 2015). Academic clinicians’ perspectives may also be attributed to their ability to have extended interactions with clients, related to guiding student learning. The opportunity to interact with clients for longer may have also contributed to academics experiencing client perspectives as a facilitator of PCC.

There is extensive discourse in the healthcare literature surrounding the concept of power balance, which encompasses various fundamental components. A critical aspect of this balance is the active engagement of clients in the care process (Castro et al. 2016; English 2022; Kitson et al. 2013; Lusk & Fater 2013). The perspective of clients was identified as a significant barrier by participating clinicians. Client perspectives encompass a multifaceted array of factors, including variations in educational backgrounds, language barriers, scepticism towards the medical healthcare system, sensitivity to specific healthcare issues, adherence to cultural taboos and alignment with traditional customs (Southwood & Van Dulm 2015). Audiologists more frequently rated client perspectives as a significant barrier as compared to SLTs, possibly because of the client population they serve. Individuals with hearing loss often contend with stigma associated with hearing loss and hearing devices and often demonstrate limited enthusiasm for engaging in their healthcare, including the acquisition and utilisation of devices (Ruusuvuori et al. 2019). Notably, audiology demands a tailored approach to fitting technology to clients’ audiological and lifestyle requirements (Timmer et al. 2023). In contrast, speech-language pathology follows a more iterative approach along an intervention continuum (Alighieri et al. 2022; Zebrowski et al. 2021), which may contribute to the differences in the perceived importance of client perspectives between these two healthcare professions.

Person-centred care is time intensive and requires clinicians to dedicate more time to understanding their clients’ specific needs and requirements (Bennett et al. 2021; Gluyas 2015). This was reflected in the responses of the participants, specifically those in the public sector and those working with geriatric populations (adult clients aged > 65 years old), where the scarcity of resources, including time and finances, was identified as a significant challenge to implementing PCC. The public sector often grapples with high client loads and inadequate financial government support, which adversely affect their capacity to deliver comprehensive PCC (Bhamjee et al. 2022; Khoza-Shangase & Mophosho 2021). Participants working with geriatric populations may experience resources as a barrier to PCC because they are working with a population that no longer earns an income but has growing medical and health-related needs that require financial resources (Souchon et al. 2020). Additionally, adults in general have more capacity to play an active role in determining the approach to their care than paediatric populations, which may explain why client perspectives were also identified as a barrier to PCC for clinicians treating geriatric populations. Interestingly, the availability of tools was noted as a barrier by most participants but was associated as a significant facilitator for clinicians working with paediatric populations. Resources for younger populations from diverse backgrounds are more readily available in South Africa than for geriatric client populations because of the push from government sectors to encourage early childhood development for improved long-term outcomes (National Planning Commission 2012).

For clinicians managing adult populations in general (19 years and older), workplace and management factors may present as barriers to PCC because of the contexts where they receive services. Adult populations are typically seen in hospital settings where management protocols and the areas that clients are seen in, for example, open wards, may restrict clinicians’ abilities to implement a PCC approach (Moore et al. 2016). Sales may also act as a barrier to PCC more so for adult populations than paediatric populations because of the acknowledged bias of individuals to be willing to incur costs for their children rather than for themselves (Dickie & Messman 2004; Monheit, Grafova & Kumar 2020).

It is evident that the personal attributes of clinicians play a vital role in fostering PCC, while age and experience influence perceptions of regulatory challenges. The time-intensive nature of PCC, coupled with resource constraints, however, poses significant challenges, especially in the public sector. Client perspectives also emerged as a critical barrier, with distinct challenges faced by AUDs. These findings collectively emphasise the multifaceted nature of PCC implementation in diverse healthcare contexts, underscoring the importance of addressing client perspectives while leveraging clinician attributes and fostering supportive workplace environments to adopt PCC successfully. Such insights into the facilitators and barriers faced by SLPs and AUDs serve as a self-reflection for clinicians, which may foster collaboration towards more effective and equitable care delivery. This introspection and acknowledgement may catalyse policy and systems changes for speech-language pathology and audiology, promoting equity and diversity in LMICs.

This study, however, has certain limitations. Before data collection, the Likert scale was expanded to 7 points, but because of the sparse data, categories collapsed back to a 5-point scale during data analysis. We acknowledge that although this could result in a loss of granularity in the data, for meaningful interpretation, it was important to ensure that the frequencies in the cells were not sparse (Field 2018). Another limitation is that the response rate was low (103/4194 = 2.5%), with the population size (4194) consisting of 1566 dually qualified clinicians (37.3%), 1548 SLPs (36.9%) and 1080 AUDs (25.8%) (hearing and acoustician professionals included), registered with the HPCSA. Low response rates for e-surveys are common and attributed to various reasons, including the rise of online surveys and information requests overwhelming respondents causing them to ignore the requests (Koen et al. 2018). Furthermore, future research should endeavour to gather more responses from clinicians in the public sector, as almost half of the respondents in this study were from the private sector. Clinicians working in public healthcare facilities experience additional factors, both facilitators and barriers, which can influence the application of a PCC approach (Khoza-Shangase & Mophosho 2018; Maphumulo & Bhengu 2019).

## Conclusion

This study explored the perceptions of South African SLPs and AUDs on implementing PCC. Clinicians’ attributes, notably age and experience, play a crucial role in fostering PCC. However, challenges arise because of the time-intensive nature of PCC and resource constraints, especially in the public sector. These findings emphasise the complex landscape of PCC implementation and the need for tailored approaches in diverse healthcare contexts.
